# Transcriptome analysis of blastoderms exposed to prolonged egg storage and short periods of incubation during egg storage

**DOI:** 10.1186/s12864-022-08463-2

**Published:** 2022-04-04

**Authors:** K. Brady, C. C. Talbot, J. A. Long, G. Welch, N. French, D. Nicholson, M. R. Bakst

**Affiliations:** 1 Animal Biosciences and Biotechnology Laboratory, BARC, ARS, USDA, 10300 Baltimore Ave. Bldg. 200, Rm. 103, Beltsville, MD 20705 USA; 2 Institute for Basic Biomedical Sciences, Johns Hopkins University School of Medicine, Baltimore, MD 21205 USA; 3 Aviagen Ltd., Newbridge, Midlothian, EH28 8SZ UK

**Keywords:** SPIDES, Egg storage, Prolonged storage, Chicken, Transcriptome, Blastoderm

## Abstract

**Background:**

Cool temperature egg storage prior to incubation is a common practice in the broiler industry; however, prolonged egg storage causes increased embryonic mortality and decreased hatchability and growth in surviving chicks. Exposing eggs to short periods of incubation during egg storage (**SPIDES**) reduces the adverse consequences of prolonged storage. SPIDES increases blastodermal cell viability by reducing apoptosis, though the counteracting mechanisms are unclear. To define the impact of prolonged storage and SPIDES, transcriptome analysis compared gene expression from blastoderms isolated from eggs exposed to the following treatments: control (CR, stored at 17 °C for 4 days), prolonged storage (NSR, stored at 17 °C for 21 days), SPIDES (SR, stored at 17 °C for 21 days with SPIDES), and incubated control (C2, stored at 17 °C for 4 days followed by incubation to HH (**Hamburger**–**Hamilton**) stage 2, used as the ideal standard development) (*n* = 3/group). Data analysis was performed using the CLC Genomics Workbench platform. Functional annotation was performed using DAVID and QIAGEN Ingenuity Pathway Analysis.

**Results:**

In total, 4726 DEGs (**differentially expressed genes**) were identified across all experimental group comparisons (q < 0.05, FPKM> 20, |fold change| > 1.5). DEGs common across experimental comparisons were involved in cellular homeostasis and cytoskeletal protein binding. The NSR group exhibited activation of ubiquitination, apoptotic, and cell senescence processes. The SR group showed activation of cell viability, division, and metabolic processes. Through comparison analysis, cellular respiration, tRNA charging, cell cycle control, and HMBG1 signaling pathways were significantly impacted by treatment and potential regulatory roles for ribosomal protein L23a (***RPL23A***) and MYC proto-oncogene, BHLH transcription factor (***MYC***) were identified.

**Conclusions:**

Prolonged egg storage (NSR) resulted in enriched cell stress and death pathways; while SPIDES (SR) resulted in enriched basic cell and anti-apoptotic pathways. New insights into DNA repair mechanisms, RNA processing, shifts in metabolism, and chromatin dynamics in relation to egg storage treatment were obtained through this study. Although egg storage protocols have been examined through targeted gene expression approaches, this study provided a global view of the extensive molecular networks affected by prolonged storage and SPIDES and helped to identify potential upstream regulators for future experiments to optimize egg storage parameters.

**Supplementary Information:**

The online version contains supplementary material available at 10.1186/s12864-022-08463-2.

## Background

Egg storage prior to incubation is an essential, operational strategy of the broiler industry, providing flexibility at the breeding, transportation, and hatchery levels. At oviposition, the embryo consists of a stage X blastoderm, with roughly 40,000 to 60,000 cells, and requires incubation temperatures above 37.5 °C for further embryonic development to transpire [1]. Embryo stage at oviposition can be impacted by strain, position in the sequence, and hen age and can influence hatchability rates [2, 3]. Between oviposition and incubation, eggs are maintained at cold temperatures (17–18 °C) to pause embryonic development and to prevent bacterial growth [4]. The incorporation of cold storage allows for the circumvention of daily egg transportation from breeding operations, increased distance allowances between breeding and hatchery operations due to environmentally controlled transport, and the management of incubator operations at the hatchery based on incubator capacity and broiler production demands. With the reliance of poultry industry operations on cold storage practices, comprehension of the impact of egg storage conditions on embryonic development, hatchability, and progeny performance are imperative for optimizing egg storage protocols and improving the operational efficiency.

Upon exposure to cold storage, embryonic development enters a temperature-induced diapause state, allowing for embryonic survival for a finite duration of time at reduced temperatures [5]. Embryonic diapause is characterized by reduced cellular metabolism and developmental arrest in the G_2_ phase of the cell cycle [6]. Entrance into a diapause state and a successful restart of development can only be achieved at particular embryonic stages, encompassing the onset of blastulation to early gastrulation (Eyal-Giladi and Kochav stages X-XIV) [7]. In addition to embryonic developmental stage requirements, ability to enter into a diapause state appears to be regulated by blastodermal cell count, pluripotency state, and cytoarchitecture; however, the regulatory pathways are not well understood [8]. Furthermore, egg component characteristics, such as albumin pH, influence the diapause state and the ability to successfully resume development [9].

Following egg lay, egg components exhibit postovipositional conformational and molecular changes to ensure protection from microbial agents and provide the ideal pH, ion gradients, and oxygen permeability to support further embryonic development. Most notably, the egg albumen undergoes rapid changes in pH and viscosity [10]. Albumen quality, particularly in terms of pH level and degree of liquefaction, is tightly linked with successful embryonic development. Increased carbon dioxide (**CO**_**2**_) and ammonia, either external to the egg in the circulating environment or produced as a byproduct of embryonic cell metabolism, have been linked with decreasing albumen pH level and increasing levels of albumen liquefaction, respectively [11, 12]. Release of CO_2_ via egg pores and ammonia clearance also impact the albumen pH level and the rate of albumen liquefaction. Moreover, albumen pH level and liquefaction appear to be linked processes [13]. Even during egg storage at temperatures below physiological zero, changes in cellular activity are observed in blastoderms leading to byproducts of blastoderm cell metabolism, ultimately contributing to egg component changes seen following oviposition [14]. Due to cell metabolism and external storage conditions, cold egg storage shifts albumen pH level from 7.6 at oviposition to 9 after 4 days of storage, with a pH of 8.2 ideal for embryonic development [15]. A rise in pH and liquefaction in the albumen appears to be advantageous up to a point, with egg storage for 2 days improving hatchability, though increased albumen pH levels are hypothesized to play a role in the negative hatchability outcomes seen with prolonged storage. In addition, prolonged storage results in reduced integrity of the perivitelline membrane, leading to reduced support for yolk sac and blastoderm development [16].

Prolonged storage (> 7 days) is associated with reduced hatchability rates, chick quality, and progeny performance [17]. Analysis of blastodermal cells following prolonged storage indicates reduced cell numbers and increased apoptotic and necrotic indexes, signifying increased cell death [18]. Additionally, an increased mitotic index is seen in blastoderms exposed to prolonged storage, potentially indicating cell cycle arrest [8]. Though apoptosis, necrosis, and cell cycle arrest have long been associated with prolonged storage, the molecular pathways regulating cell death, the cell cycle, and interplay between remains unclear. The addition of short periods of incubation during egg storage (**SPIDES**) has been previously shown to mitigate the negative effects associated with prolonged storage, with SPIDES treatment recovering a large percentage of the reduced hatchability rates seen with prolonged storage [19]. Additionally, there is evidence that SPIDES treatment results in improved chick quality, with body weights similar to those seen in chicks derived from unstored eggs and improved initial growth curves [20]. Though the benefits of SPIDES treatment are apparent from an applied setting, the cellular and molecular mechanisms leading to the reduction of physiological cellular stress are not well understood. It is hypothesized that SPIDES potentially resets the embryonic diapause mechanisms with each period of incubation and/or allows for basic cell metabolism functions to occur during the incubation periods, limiting the activation of apoptosis and necrosis pathways characteristic of prolonged storage.

While prolonged storage and SPIDES treatment have been associated with differences in hatchability and progeny performance as detailed above, the molecular mechanisms regulating apoptosis and necrosis in embryos exposed to prolonged storage and mitigation of the negative effects of prolonged storage in embryos exposed to SPIDES are not defined. Additionally, early embryonic metabolism is impacted by egg storage type and duration and may play a role in influencing egg component composition, which in turn, impacts the ability for blastoderms to resume embryonic development when exposed to incubation conditions. In order to gain a deeper understanding of molecular dynamics impacted by egg storage protocols, a transcriptomics approach was used to compare global gene expression of blastoderms exposed to four storage and incubation combinations: control (**CR**, stored for 4 days at 17 °C), incubated control (**C2**, stored for 4 days at 17 °C then incubated to HH stage 2, used as an example of normal blastoderm development), prolonged storage (**NSR**, stored for 21 days at 17 °C), and SPIDES (**SR**, stored for 21 days at 17 °C with three 4-h incubation periods). Differential gene expression was determined between the following groups: (1) CR and C2 to determine normal blastoderm development, (2) CR and NSR to determine the effects of prolonged storage, (3) NSR and SR to compare the impact of prolonged storage and SPIDES treatment, and (4) C2 and SR to determine the impact of SPIDES treatment on blastoderm development. Insights from the obtained data will help to understand the molecular mechanisms regulating hatchability differences among eggs exposed to prolonged storage and SPIDES treatment, which may allow for optimization of current long-term egg storage protocols.

## Results

### Overview of DEGs

A total of 1.7 billion reads were obtained across the twelve samples sequenced. On average, 142,974,782 reads were obtained per sample, with 89.8% of reads mapping in pairs, 3.6% of reads mapping in broken pairs, and 6.6% of reads not mapping to the chicken genome (Additional Fig. 1A). Of the reads mapped in pairs, 79.24% of reads mapped to exonic regions, 8.16% of reads mapped to intronic regions, and 12.60% of reads mapped to intergenic regions (Additional Fig. 1B). The number of raw reads, reads mapped in pairs, reads mapped in broken pairs, and reads that did not map to the chicken genome did not differ between experimental groups (*p* > 0.05). Additionally, the number of reads mapping to exonic, intronic, and intergenic regions did not differ between experimental groups (*p* > 0.05). Principle component analysis revealed clustering by experimental group with clear separation between groups (Additional Fig. 2A). Clustering by experimental group was also seen following heat map analysis of the mapped reads across all of samples (Additional Fig. 2B). Lastly, gene expression results obtained through RNA sequencing were confirmed using quantitative PCR methods, revealing similar mRNA expression profiles for the 10 genes examined (Additional Fig. 3A and B).

**Fig. 1:**
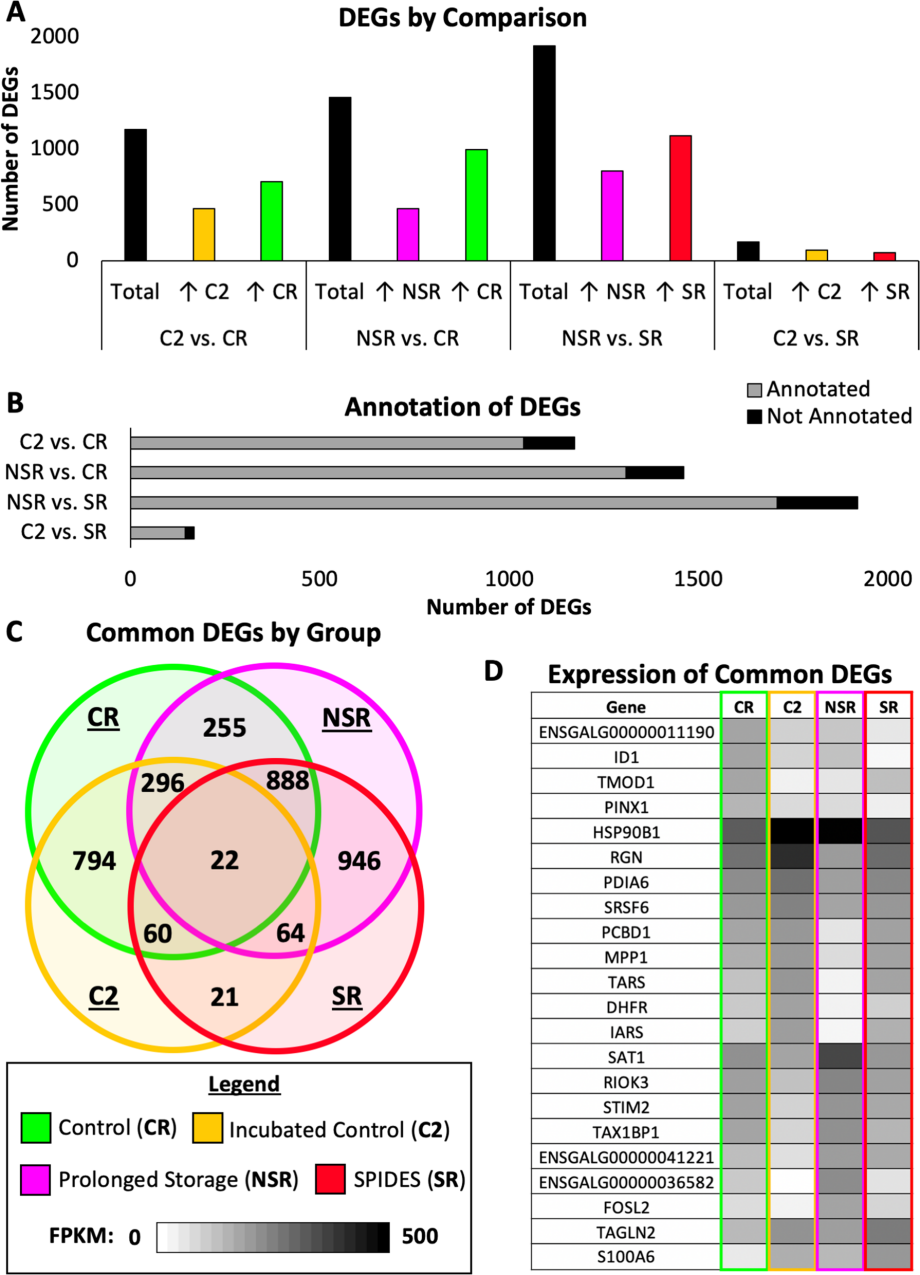
Characterization of differentially expressed genes (DEGs). (**A**) Numbers of total differentially expressed genes (DEGs) for each comparison, with the number of DEGs up-regulated (↑) in each experimental group (FPKM > 20, |fold change| > 1.5, q < 0.05). (**B**) The portion of DEGs that are annotated and unannotated in the chicken genome (GRCg6a; ENSEMBL annotation release 101) (FPKM > 20, |fold change| > 1.5, q < 0.05). (**C**) Venn diagram showing the number of common DEGs among each experimental comparison (FPKM > 20, |fold change| > 1.5, q < 0.05). (**D**) Heat map showing the gene name and expression profile (provide as FPKM) across control (CR), incubated control (C2), prolonged storage (NSR), and SPIDES (SR) groups for each of the DEGs common to all four experimental comparisons

After performing differential expression analysis, 4726 DEGs were identified across all experimental group comparisons, with 1177 DEGs identified between C2 and CR experimental groups, 1468 DEGs identified between NSR and CR experimental groups, 1915 DEGs identified between NSR and SR experimental groups, and 167 DEGs identified between C2 and SR experimental groups (q < 0.05, |fold change| > 1.5, FPKM (**fragments per kilobase of exon per million reads mapped**) > 20) (Fig. 1A, see Additional file 5 for top expressed transcripts). The highest number of DEGs were identified between the NSR and SR experimental groups, whereas the lowest of number of DEGs were identified between the C2 and SR experimental groups, indicating the highest degree of dissimilarity among the NSR and SR group transcriptomes and the highest degree of similarity among the C2 and SR group transcriptomes. The proportion of unannotated genes did not differ between comparisons, with roughly 10% of the total DEGs unannotated (Fig. 1B). A portion of the DEGs identified for each experimental comparison showed overlap across comparisons (Fig. 1C). A total of 22 DEGs were common to all four experimental comparisons, with 4 genes in the CR experimental group, 9 genes in the C2 experimental group, 7 genes in the NSR experimental group, and 2 genes in the SR experimental group exhibiting the highest expression level compared to the other experimental groups (Fig. 1D). Functional annotation analysis of the common DEGs showed enrichment of cellular and biological processes, regulation, and homeostasis (Table 1). Additionally, enrichment of processes involving tetrahydrobiopterin, pteridine, and tropomyosin was exhibited. The common DEGs showed enrichment for localization to extracellular, cell membrane, and cytoplasmic regions.

**Table 1:** Functional annotation analysis of DEGs common to all experimental comparisons

**Biological Process (BP)**				
**Term**	**Count**	***P*** **Value**	**Enriched**	**Genes**
regulation of cellular process	13	0.0049	1.69	TMOD1, MPP1, PDIA6, SAT1, FOSL2, HSP90B1, DHFR, STIM2, ID1, TAX1BP1, S100A6, RGN, PCBD1
regulation of biological process	13	0.0085	1.60	TMOD1, MPP1, PDIA6, SAT1, FOSL2, HSP90B1, DHFR, STIM2, ID1, TAX1BP1, S100A6, RGN, PCBD1
tetrahydrobiopterin biosynthetic process	2	0.0086	214.14	DHFR, PCBD1
tetrahydrobiopterin metabolic process	2	0.0086	214.14	DHFR, PCBD1
pteridine-containing compound biosynthetic process	2	0.0158	116.81	DHFR, PCBD1
biological regulation	13	0.0179	1.50	TMOD1, MPP1, PDIA6, SAT1, FOSL2, HSP90B1, DHFR, STIM2, ID1, TAX1BP1, S100A6, RGN, PCBD1
cellular homeostasis	4	0.0190	6.28	STIM2, RGN, PDIA6, HSP90B1
pteridine-containing compound metabolic process	2	0.0257	71.38	DHFR, PCBD1
cellular metal ion homeostasis	3	0.0409	8.53	STIM2, RGN, HSP90B1
**Cellular Component (CC)**				
**Term**	**Count**	***P*** **Value**	**Enriched**	**Genes**
extracellular exosome	6	0.0202	3.21	TAX1BP1, S100A6, TARS, PCBD1, PDIA6, HSP90B1
extracellular vesicle	6	0.0206	3.19	TAX1BP1, S100A6, TARS, PCBD1, PDIA6, HSP90B1
extracellular organelle	6	0.0207	3.19	TAX1BP1, S100A6, TARS, PCBD1, PDIA6, HSP90B1
extracellular region	7	0.0281	2.49	TAX1BP1, S100A6, RGN, TARS, PCBD1, PDIA6, HSP90B1
cytoplasm	11	0.0287	1.61	MPP1, TMOD1, STIM2, ID1, S100A6, RGN, TARS, PCBD1, PDIA6, SAT1, HSP90B1
membrane-bounded vesicle	6	0.0439	2.64	TAX1BP1, S100A6, TARS, PCBD1, PDIA6, HSP90B1
**Molecular Function (MF)**				
**Term**	**Count**	***P*** **Value**	**Enriched**	**Genes**
tropomyosin binding	2	0.0136	135.43	TMOD1, S100A6

The top biological process (**BP**), cellular component (**CC**), and molecular function (**MF**) gene ontology (**GO**) terms for differentially expressed genes (**DEGs**) common across all experimental comparisons and the associated count, *p*-value, fold enrichment, and involved genes are shown (count > 2, *p*-value < 0.05, fold enrichment > 1.5)

### Gene ontology (GO) and KEGG analysis

**Fig. 2:**
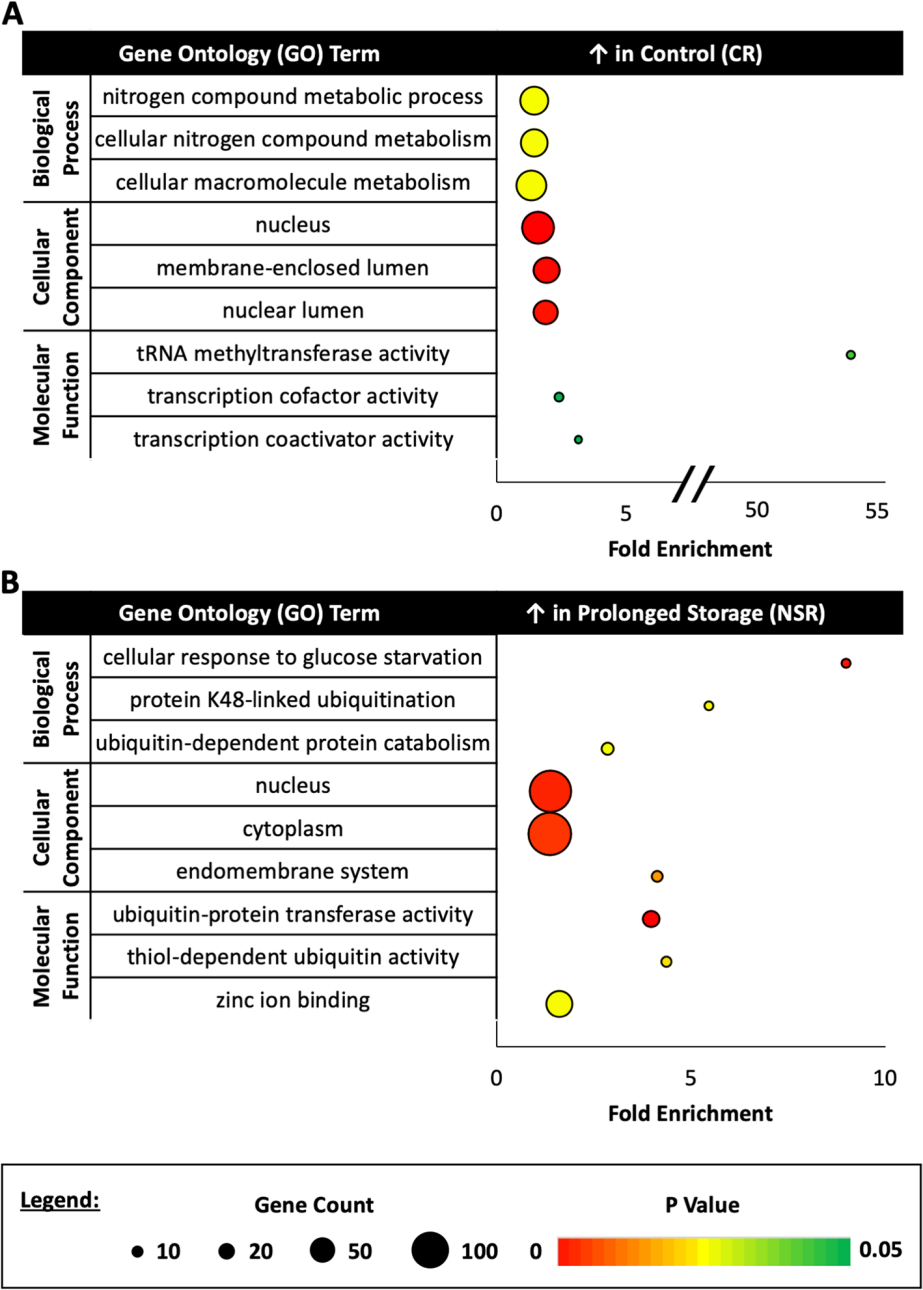
Gene ontology (GO) analysis of control (CR) and prolonged storage (NSR) experimental groups. (**A**) The top three biological process (BP), cellular component (CC), and molecular function (MF) gene ontology (GO) terms for differentially expressed genes (DEGs) upregulated in the control (CR) group in both experimental comparisons (count > 2, *p*-value < 0.05, fold enrichment > 1.5). (**B**) The top three BP, CC, and MF GO terms for DEGs upregulated in the prolonged storage (NSR) group in both experimental comparisons (count > 2, p-value < 0.05, fold enrichment > 1.5)

**Fig. 3:**
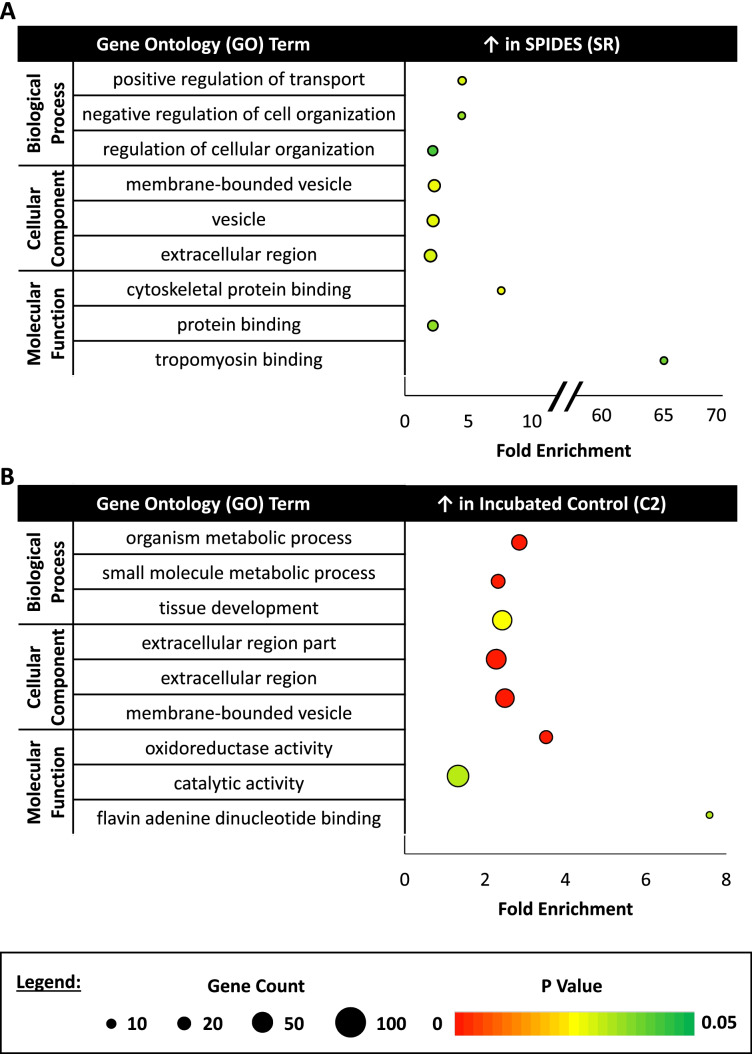
Gene ontology (GO) analysis of SPIDES (SR) and incubated control (C2) experimental groups. (**A**) The top three biological process (BP), cellular component (CC), and molecular function (MF) gene ontology (GO) terms for differentially expressed genes (DEGs) upregulated in the SPIDES (SR) group in both experimental comparisons (count > 2, p-value < 0.05, fold enrichment > 1.5). (**B**) The top three BP, CC, and MF GO terms for DEGs upregulated in the incubated control (C2) group in both experimental comparisons (count > 2, p-value < 0.05, fold enrichment > 1.5)

For each experimental group, upregulated DEGs common to that experimental group across both comparisons were subjected to gene ontology (**GO**) analysis through DAVID. The CR group displayed increased expression for genes associated with nitrogen and macromolecule metabolism as well as tRNA methyltransferase and transcription cofactor/coactivation activity, with nuclear localization enrichment (Fig. 2A). In the NSR group, upregulation of genes related to protein ubiquitination, glucose deficiency, and zinc ion binding was seen, with localization enrichment distributed across the cell components (Fig. 2B). The SR group, exhibited enriched gene expression linked to cellular transport/organization and binding of cytoskeletal components, with enriched localization in vesicle and extracellular regions (Fig. 3A). Lastly, the C2 group showed upregulated gene expression correlated with cell metabolism, tissue development, and redox reaction activity, with localization enriched to extracellular and vesicle regions (Fig. 3B).

**Fig. 4:**
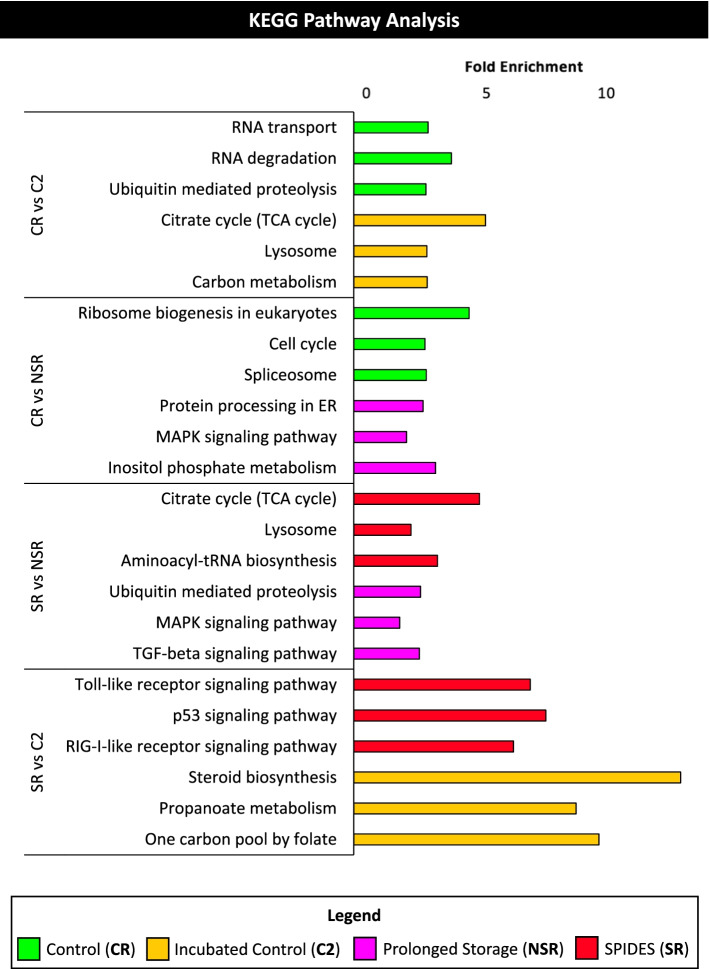
KEGG pathway analysis of experimental comparisons. For each experimental comparison, the top three pathways upregulated in each group (count > 2, p-value < 0.05, fold enrichment > 1.5)

KEGG pathway analysis was performed on DEGs for each experimental comparison to determine biological pathway enrichment (Fig. 4). Upregulated DEGs in the CR group were associated with RNA transport and degradation, ubiquitin mediated proteolysis, ribosome and spliceosome activity, and the cell cycle. Within the NSR group, upregulated DEGs were related to ubiquitin mediated proteolysis, MAPK signaling, protein processing in the endoplasmic reticulum, TGF-beta signaling, and inositol phosphate metabolism. In the SR group, upregulated DEGs were associated with toll-like receptor, p53, and RIG-I-like receptor signaling. Additionally, the SR group exhibited gene expression consistent with enrichment of the TCA cycle and aminoacyl-tRNA biosynthesis activity. The C2 group showed increased expression of genes related to propanoate and carbon metabolism, steroid biosynthesis, and activity of metabolic and lysosome pathways.

### Blastoderm development (C2 vs CR)

**Fig. 5:**
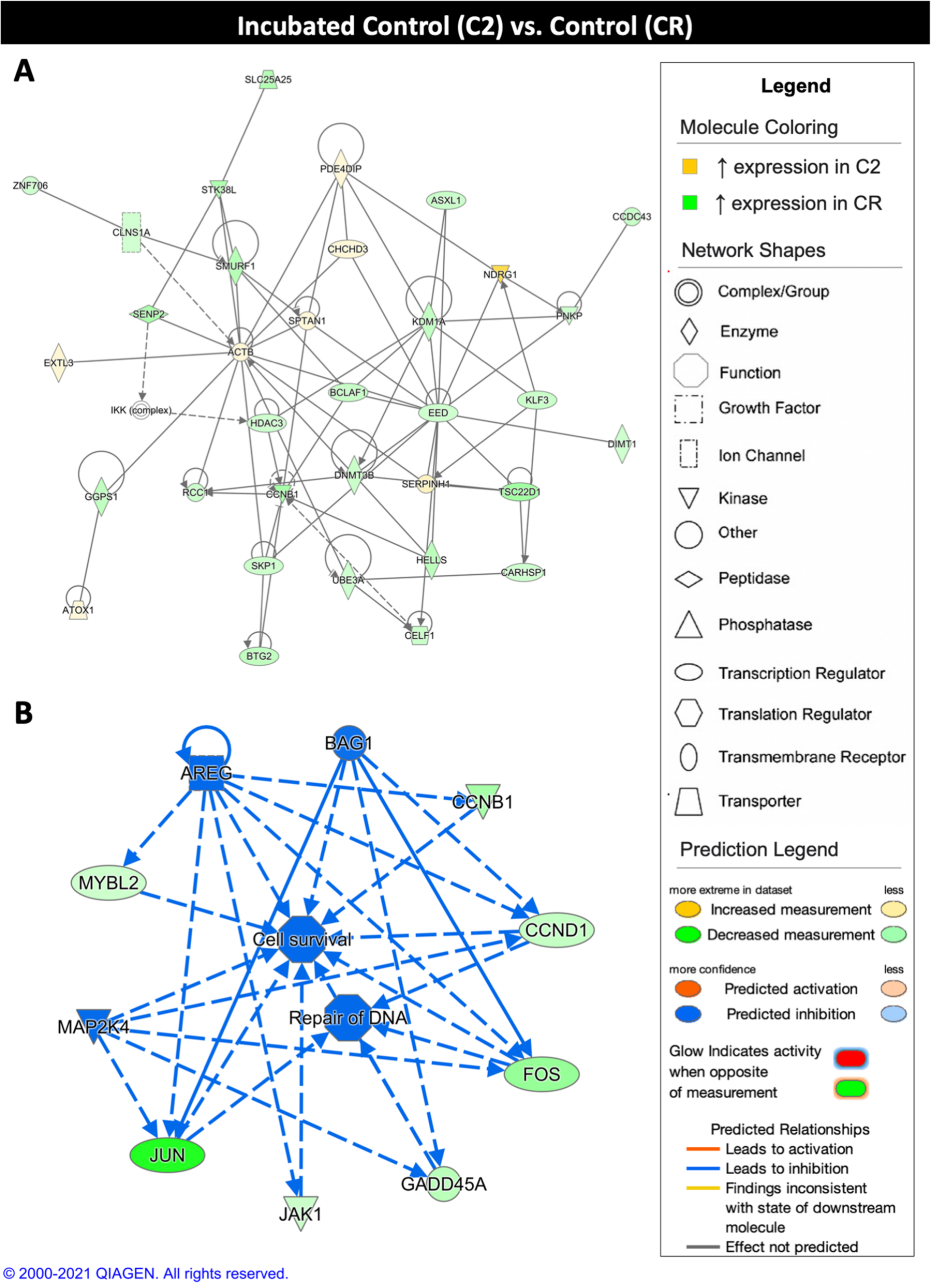
Ingenuity pathway core analysis (IPA) of incubated control (C2) and control (CR) groups. Ingenuity® Pathway Analysis (Qiagen, Valencia, CA) was used to analyze differentially expressed genes (DEGs) to biologically interpret expression data. Copyright permission from Qiagen has been obtained for use of the images presented. (**A**) The top network obtained through pathway analysis of DEGs between incubated control (C2) and control (CR) groups (network score = 48, top function = organismal survival) (FPKM > 20, p-value < 0.05, |fold change| > 1.5). (**B**) The top regulator effects network obtained through pathway analysis of DEGs between C2 and control CR groups (consistency score = 12.2) (FPKM > 20, p-value < 0.05, |fold change| > 1.5)

Further functional annotation of the DEGs resulting from the comparison of C2 and CR experimental groups showed networks associated with organism survival, RNA post-transcriptional modifications, and small molecular biochemistry (Table 2). Upstream analysis revealed MYC proto-oncogene, BHLH transcription factor (**MYC**) and cyclin F (**CCNF**), which function in cell cycle progression and ubiquitination, respectively, as the top regulators predicted to be inhibited in the C2 group and both show decreased expression in the C2 group in contrast to the CR group. Furthermore, SRY (Sex Determining Region Y)-Box 17 (**SOX17**), a regulator of embryonic development, was the top regulator predicted to be activated in in the C2 group and exhibits increased expression levels in the C2 group than in the CR group. The top scoring network shows upregulation expression of genes with roles in organismal death, reduced transcription, and senescence of cells in the CR group compared to the C2 group (Fig. 5A). The top scoring regulator effect network identifies amphiregulin (**AREG**), BAG cochaperone 1 (**BAG1**), and mitogen-activated protein kinase kinase 4 (**MAP2K4**) as potentially inhibited regulators, leading to inhibition of cell survival and repair of DNA in the C2 group in comparison with the CR group (Fig. 5B).

**Table 2:** Summary of Ingenuity Pathway Analysis results for C2 vs. CR comparison

**Network Score**	**Top Functions**				
48	Organismal Survival				
45	RNA Post-Transcriptional Modification, Small Molecule Biochemistry				
41	RNA Post-Transcriptional Modification				
**Regulators in Network**		**Score**	**Targets**	**Functions**	
AREG, BAG1, MAP2K4		12.2	7	Cell survival, DNA repair	
CXCL12, FSH, GPER1, GH, HGF, MAP2K1/2		5.9	15	mRNA expression, Immortalization	
ANGPT2, PTPN3, Rb		3.1	13	Microtubule dynamics, Senescence of cells	
**Upstream Regulator**	**Fold**	**Molecule Type**		**Activation z-score**	***P*** **Value**
MYC	−1.564	transcription regulator		−2.606	1.31E-12
CXCL12	−1.671	cytokine		−2.523	4.37E-02
CCND1	−1.901	transcription regulator		−2.274	1.18E-05
S100A6	2.441	transporter		−2.236	1.33E-02
SOX17	2.573	transcription regulator		2.393	1.22E-03
**Master Regulator**	**Fold**	**Molecule Type**		**Activation z-score**	***P*** **Value**
CCNF	−1.866	other		−3.43	3.50E-03
KDM5B	1.588	transcription regulator		−2.949	7.00E-04
TFDP2	−1.877	transcription regulator		−2.832	5.00E-04
MNAT1	−1.798	other		−2.554	1.80E-02
TLK2	−1.538	kinase		−2.236	2.22E-02

The top three networks (network score > 40) and top three regulator effect networks (consistency score > 0, target number > 3, |z-score| > 2, *p*-value < 0.05) are shown. The top five activated (z-score > 2) and inhibited (z-score < − 2) upstream regulators and master regulators in causal networks are shown (|fold change| > 1.5, |z-score| > 2, *p*-value < 0.05). Only upstream regulators and master regulators exhibiting differential expression between C2 and CR groups are displayed

### Impact of prolonged storage (NSR vs CR)

**Fig. 6:**
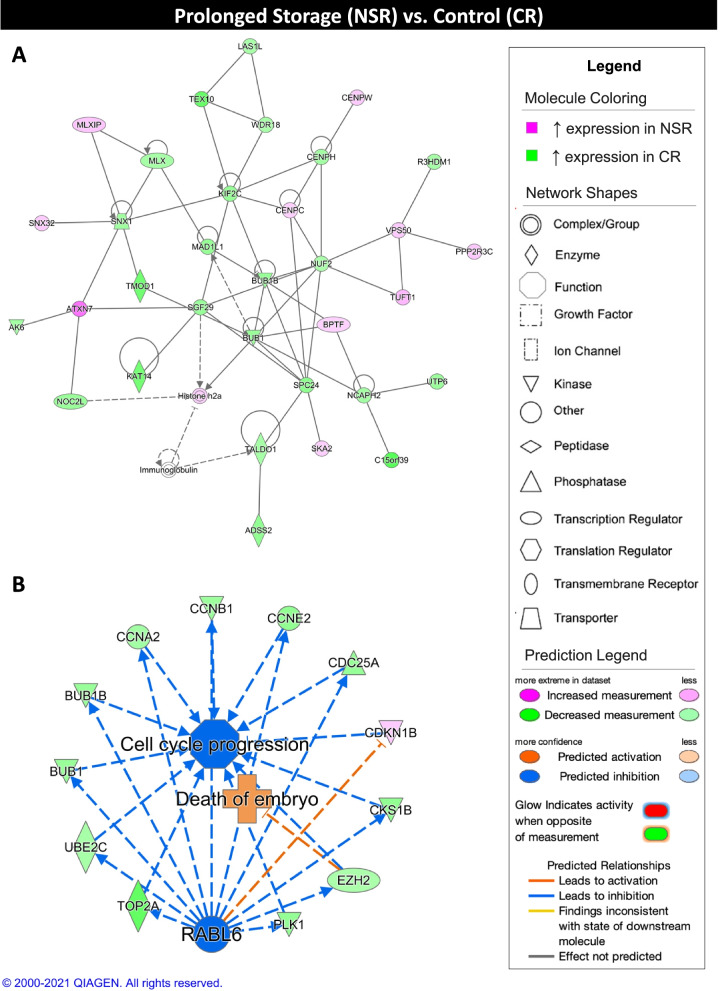
Ingenuity pathway core analysis (IPA) of prolonged storage (NSR) and control (CR) groups. Ingenuity® Pathway Analysis (Qiagen, Valencia, CA) was used to analyze differentially expressed genes (DEGs) to biologically interpret expression data. Copyright permission from Qiagen has been obtained for use of the images presented. (**A**) The top network obtained through pathway analysis of DEGs between prolonged storage (NSR) and control (CR) groups (network score = 42, top functions = cell cycle, cellular assembly and organization, DNA replication, recombination, and repair) (FPKM > 20, *p*-value < 0.05, |fold change| > 1.5). (**B**) The top regulator effects network obtained through pathway analysis of DEGs between NSR and CR groups (consistency score = 13) (FPKM > 20, p-value < 0.05, |fold change| > 1.5)

Comparison of the NSR and CR experimental groups produced networks associated with cell cycle and assembly, protein synthesis, DNA dynamics, embryo death (Table 3). Upstream analysis revealed twinkle mtDNA helicase (**TWNK**) and baculoviral IAP repeat containing 5 (**BIRC5**), which have mtDNA replication and antiapoptotic roles, respectively, as the top inhibited regulators in the NSR group. Additionally, both show decreased expression in the NSR group compared to the CR group. On the other hand, transcription factor 3 (**TCF3**), a transcriptional repressor, and BRCA1 associated RING domain 1 (**BARD1**), which controls the cell cycle in response to DNA damage, were the top activated regulators in the NSR group and both show increased expression levels in the NSR group. In the top scoring network, the CR group exhibited gene expression associated with cell cycle progression and morphology of the cytoskeleton (Fig. 6A). In the top scoring regulator effect network, RAB, member RAS oncogene family like 6 (**RABL6**) was identified as a potentially inhibited regulator, resulting in inhibition of cell cycle progression and activation of embryo death in the NSR group in comparison with the CR group (Fig. 6B).

**Table 3:** Summary of Ingenuity Pathway Analysis results for NSR vs. CR comparison

**Network Score**	**Top Functions**				
42	Cell Cycle, Cellular Assembly and Organization, DNA Replication, Recombination, and Repair				
42	Protein Synthesis				
42	Cell Cycle				
**Regulators in Network**		**Score**	**Targets**	**Functions**	
RABL6		13	12	Cell cycle progression, Embryo death	
CDKN1B, E2F2, LIN9, MYBL2, RABL6, RBL1, TASP1		12	37	G1/S phase transition	
E2F2, LIN9, mir-145		10.4	24	Cell cycle progression, Organismal death	
**Upstream Regulator**	**Fold**	**Molecule Type**		**Activation z-score**	***P*** **Value**
TWNK	−1.811	enzyme		−3	5.17E-06
ACTL6A	−1.699	other		−2.236	0.00805
GNL3	−1.713	other		−2.236	0.0148
TCF3	1.645	transcription regulator		2.613	8.18E-06
**Master Regulator**	**Fold**	**Molecule Type**		**Activation z-score**	***P*** **Value**
BIRC5	−1.985	other		−8.472	0.0001
SRSF2	−2.236	transcription regulator		−6.146	0.0001
PDCD4	2.187	other		−5.543	0.0001
TFDP1	−1.871	transcription regulator		−5.489	0.0001
CCNE1	−2.423	transcription regulator		−5.384	0.0001
COPS2	−1.639	other		5.514	0.0001
APLP2	1.835	other		5.846	0.0001
HEXIM1	−1.604	transcription regulator		5.996	0.0001
UCHL1	−1.581	peptidase		6.068	0.0001
BARD1	1.961	transcription regulator		6.072	0.0001

The top three networks (network score > 40) and top three regulator effect networks (consistency score > 0, target number > 3, |z-score| > 2, *p*-value < 0.05) are shown. The top five activated (z-score > 2) and inhibited (z-score < −2) upstream regulators and master regulators in causal networks are shown (|fold change| > 1.5, |z-score| > 2, *p*-value < 0.05). Only upstream regulators and master regulators exhibiting differential expression between NSR and CR groups are displayed

### Impact of SPIDES treatment (NSR vs SR)

**Fig. 7:**
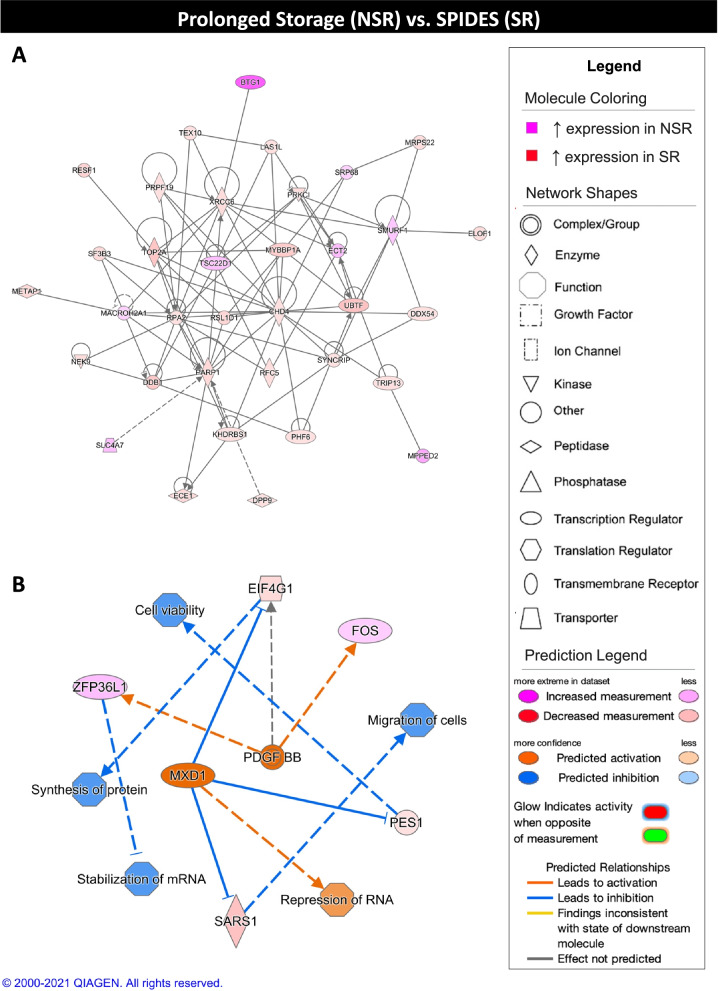
Ingenuity pathway core analysis (IPA) of prolonged storage (NSR) and SPIDES (SR) groups. Ingenuity® Pathway Analysis (Qiagen, Valencia, CA) was used to analyze differentially expressed genes (DEGs) to biologically interpret expression data. Copyright permission from Qiagen has been obtained for use of the images presented. (**A**) The top network obtained through pathway analysis of DEGs between prolonged storage (NSR) and SPIDES (SR) groups (network score = 43, top function = carbohydrate metabolism, nucleic acid metabolism, small molecule biochemistry) (FPKM > 20, p-value < 0.05, |fold change| > 1.5). (**B**) The top regulator effects network obtained through pathway analysis of DEGs between NSR and SR groups (consistency score = 3.3) (FPKM > 20, p-value < 0.05, |fold change| > 1.5)

In the NSR and SR experimental group comparison, networks related to cell viability, metabolism, gene expression, and DNA dynamics resulted from analysis of DEGs (Table 4). Similar to the NSR and CR experimental group comparison, TWNK was found to be a top inhibited regulator in the NSR group and to be downregulated in the NSR group compared to the SR group. In addition, proliferation-associated 2G4 (**PA2G4**), which is involved in rRNA processing, was predicted to be inhibited in and showed decreased expression in the NSR group. Conversely, TSC22 domain family member 1 (**TSC22D1**), a transcription factor with roles in apoptosis, was the top activated regulator in the NSR group and showed increased expression in the NSR group compared to the SR group. For the top scoring network, SR group gene expression was related to repair of DNA and transcription, processing, and expression of RNA (Fig. 7A). The top scoring regulator effect network showed activation of platelet derived growth factor subunit B (**PDGF BB**) and MAX dimerization protein 1 (**MXD1**) with resulting inhibition of cell viability, protein synthesis, mRNA stabilization, and cell migration as well as with activation of RNA repression in the NSR group compared to the SR group (Fig. 7B).

**Table 4:** Summary of Ingenuity Pathway Analysis results for NSR vs. SR comparison

**Network Score**	**Top Functions**			
43	Cell Death and Survival, Cellular Function and Maintenance, Embryonic Development			
43	Carbohydrate Metabolism, Nucleic Acid Metabolism			
43	Cell Cycle, Cell Morphology, DNA Replication, Recombination, and Repair			
**Regulators in Network**	**Score**	**Targets**	**Functions**	
MXD1, PDGF BB	3.3	5	Cell viability, Migration of cells, Synthesis of protein, mRNA stabilization, RNA repression	
MAP2K4, MXD1, PDGF BB	2.5	13	Expression of mRNA, rRNA modification	
PCGEM1	2	9	Cell proliferation	
**Upstream Regulator**	**Fold**	**Molecule Type**	**Activation z-score**	***P*** **Value**
TWNK	−1.604	enzyme	−2.72	5.77E-07
CCNK	1.53	kinase	−2.433	0.0373
H2AX	1.84	transcription regulator	−2.418	0.00207
SAFB	−1.876	other	−2.333	0.0301
**Master Regulator**	**Fold**	**Molecule Type**	**Activation z-score**	***P*** **Value**
PA2G4	−1.52	transcription regulator	−4.518	0.0001
PDCD4	1.749	other	−4.5	0.0001
CHAF1A	−1.649	other	−4.205	0.0001
HMGA1	−1.608	transcription regulator	−4.106	0.0001
DNAJB1	1.756	transcription regulator	4.643	0.0001
TADA3	−2.638	transcription regulator	4.687	0.0001
BARD1	2.675	transcription regulator	4.692	0.0001
CBL	2.257	transcription regulator	4.739	0.0026
TSC22D1	2.081	transcription regulator	4.826	0.0001

The top three networks (network score > 40) and top three regulator effect networks (consistency score > 0, target number > 3, |z-score| > 2, *p*-value < 0.05) are shown. The top five activated (z-score > 2) and inhibited (z-score < −2) upstream regulators and master regulators in causal networks are shown (|fold change| > 1.5, |z-score| > 2, *p*-value < 0.05). Only upstream regulators and master regulators exhibiting differential expression between NSR and SR groups are displayed

### Impact of SPIDES on blastoderm development (C2 vs SR)

**Fig. 8:**
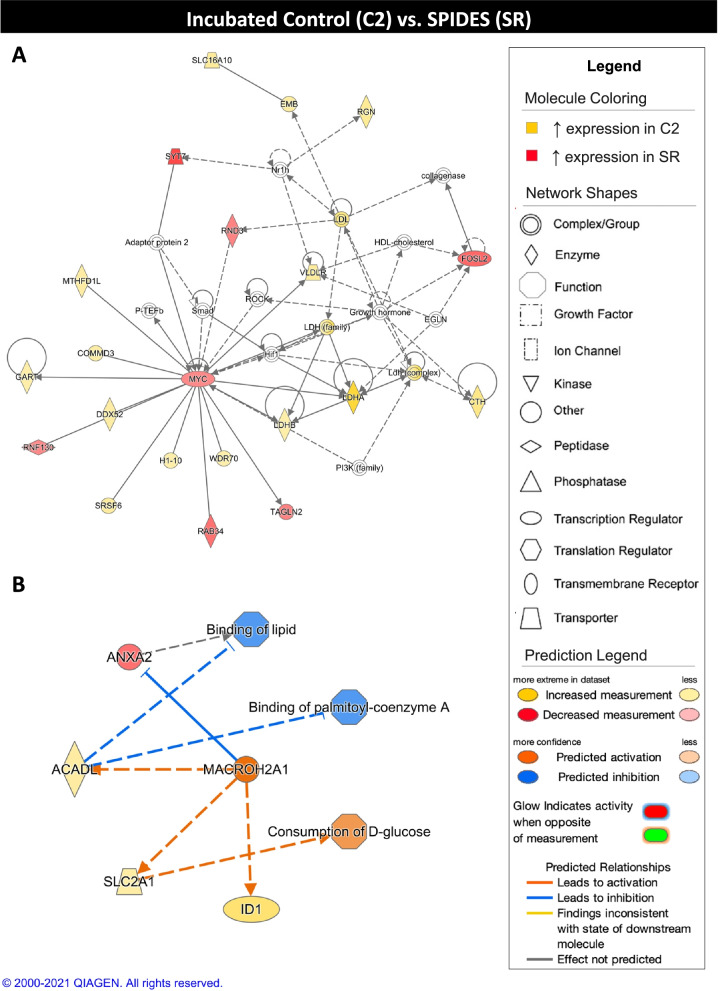
Ingenuity pathway core analysis (IPA) of incubated control (C2) and SPIDES (SR) groups. Ingenuity® Pathway Analysis (Qiagen, Valencia, CA) was used to analyze differentially expressed genes (DEGs) to biologically interpret expression data. Copyright permission from Qiagen has been obtained for use of the images presented. (**A**) The top network obtained through pathway analysis of DEGs between incubated control (C2) and SPIDES (SR) groups (network score = 42, top function = carbohydrate metabolism, energy production, small molecule biochemistry) (FPKM > 20, p-value < 0.05, |fold change| > 1.5). (**B**) The top regulator effects network obtained through pathway analysis of DEGs between C2 and SR groups (consistency score = 3) (FPKM > 20, p-value < 0.05, |fold change| > 1.5)

When the C2 and SR experimental group were compared, networks associated with energy metabolism resulted from DEG functional annotation analysis (Table 5). NFKB inhibitor alpha (**NFKBIA**), which plays a role in apoptosis, was the top inhibited regulator in the C2 group and showed decreased expression in the C2 group compared to the SR group. X-box binding protein 1 (**XBP1**), which is involved in early embryonic development, was the top activated regulator in the C2 group and showed increased expression in the C2 group compared to the SR group. The top scoring network showed C2 group gene expression consistent with upregulation of glycolysis (Fig. 8A). The top scoring regulator effect network showed activation of macroH2A.1 histone (**MACROH2A1**) associated with inhibition of lipid and palmitoyl-coenzyme A binding and activation of D-glucose consumption in the C2 group when compared to the SR group (Fig. 8B).

**Table 5:** Summary of Ingenuity Pathway Analysis results for C2 and SR comparison

**Network Score**	**Functions**			
42	Carbohydrate Metabolism, Energy Production			
**Regulators in Network**	**Score**	**Targets**	**Functions**	
MACROH2A1	3	4	Consumption of D-glucose, Binding of lipid, Binding of palmitoyl-coenzyme A	
**Upstream Regulator**	**Fold**	**Molecule Type**	**Activation z-score**	***P*** **Value**
NFKBIA	−1.596	transcription regulator	−2.377	0.000129
MYC	−1.511	transcription regulator	2.359	1.79E-09
XBP1	1.878	transcription regulator	2.593	1.86E-05

The top three networks (network score > 40) and top three regulator effect networks (consistency score > 0, target number > 3, |z-score| > 2, *p*-value < 0.05) are shown. The top five activated (z-score > 2) and inhibited (z-score < − 2) upstream regulators and master regulators in causal networks are shown (|fold change| > 1.5, |z-score| > 2, *p*-value < 0.05). Only upstream regulators and master regulators exhibiting differential expression between C2 and SR groups are displayed

### Comparison analysis

**Fig. 9:**
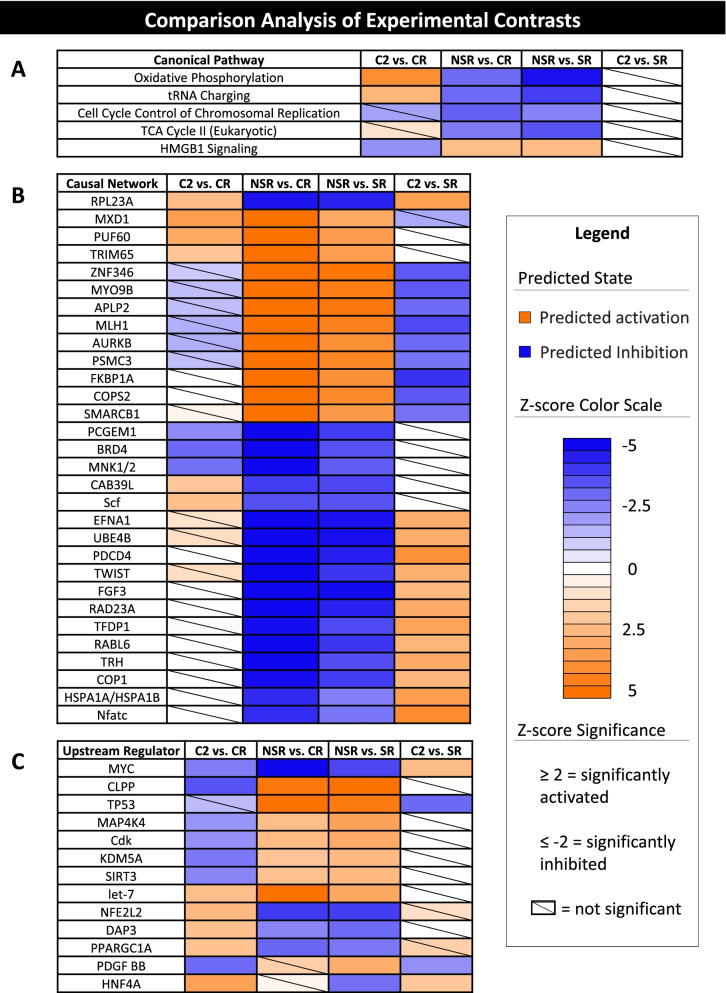
Ingenuity pathway comparison analysis (IPA) of experimental groups. Ingenuity® Pathway Analysis (Qiagen, Valencia, CA) was used to compare the core analysis results for each experiment comparison. Activity of canonical pathways, master regulators from causal networks, and upstream regulators common to at least three of the four experimental comparisons are presented (|z-score| > 2, p-value < 0.05)

**Fig. 10:**
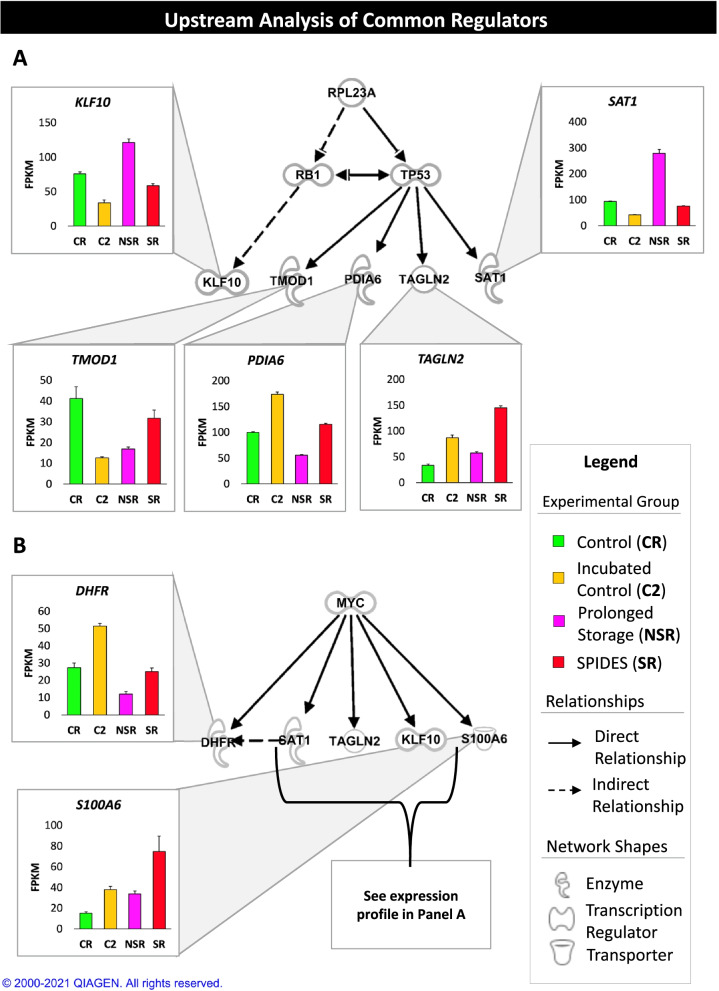
Ingenuity pathway upstream analysis (IPA) of common gene expression regulators. A detailed view of the one master regulator (RPL23A, Ribosomal Protein L23a) and one upstream regulator (MYC, MYC proto-oncogene, bHLH transcription factor) that showed significant activity in all four comparisons. FPKM values for each experimental group are shown for each regulator and the downstream targets of the regulator, with letters to denote significant expression differences between the experimental groups

The comparison tool in Ingenuity Pathway Analysis was used to determine common canonical pathways, upstream regulators, and master regulators in causal networks among the experimental comparisons (Fig. 9). Oxidative phosphorylation, tRNA charging, chromosomal replication, and the TCA cycle were predicted to be inhibited in the NSR group, whereas HMGB1 signaling was predicted to be activated. Several master and upstream regulators, with roles in cell cycle regulation, apoptosis, ubiquitination, and negative regulation of transcription, showed predicted activation in the NSR group compared with the SR and CR groups. Conversely, master and upstream regulators with roles in cell proliferation, cellular respiration, chromatin remodeling, and positive regulation of transcription, showed predicted inhibition in the NSR group compared with the SR and CR groups. The master regulator ribosomal protein L23a (**RPL23A**), which promotes tumor protein p53 (**TP53**) degradation, showed significant activity across all experimental comparisons, with predicted activation in the C2 group and inhibition in the NSR group. Downstream targets of RPL23A exhibit differential expression among the experimental groups (Fig. 10A). MYC (cell cycle progression) was identified as a common upstream regulator in all experimental comparisons, with predicted inhibition in the NSR group and with the C2 group exhibiting differential predicted activity in comparisons with the CR and SR groups. Similar to RPL23A, downstream targets of MYC exhibit differential expression among the experimental groups (Fig. 10B).

## Discussion

**Fig. 11:**
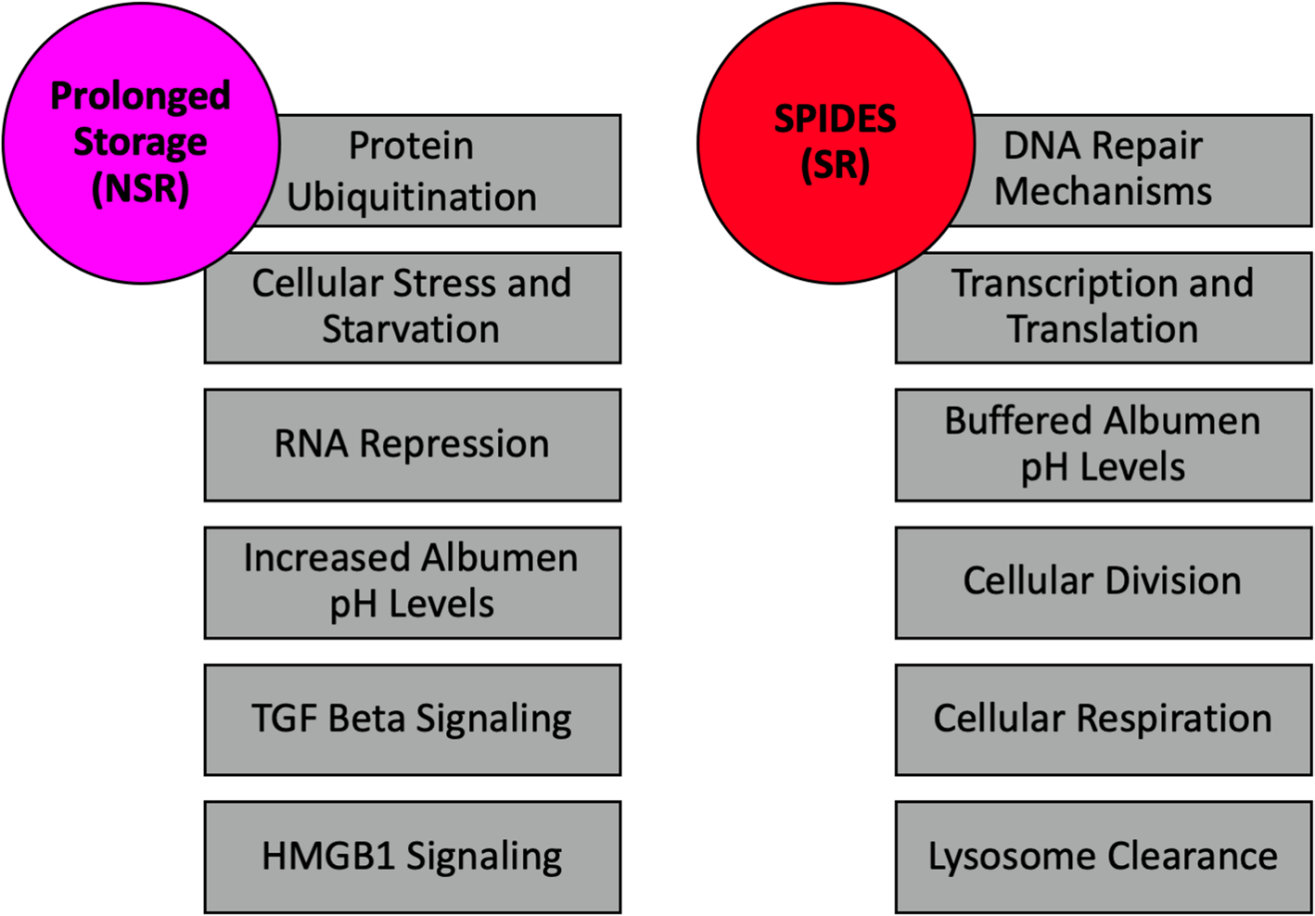
Summary of the pathways upregulated by prolonged storage (NSR) and SPIDES (SR) treatments

This study was the first transcriptomics study to examine the impact of SPIDES treatment on embryonic development and viability and compare global gene expression to blastoderms exposed to standard cold storage and prolonged cold storage as well as early embryos of the same developmental age. Through this analysis, novel insights into shifts in cellular metabolism, gene regulation, cell cycle control, and cell death were revealed. Several trends were noted based on transcriptome analysis: (1) embryo metabolism, though reduced with cold storage, may play a role in embryonic gene expression through DNA methylation mechanisms and in influencing egg component composition through metabolic byproducts, such as reactive oxygen species (**ROS**), ammonia, and CO_2_, (2) cell viability issues, ultimately leading to cell apoptosis, which may reduce blastoderm cell numbers below the threshold required for resumption of development, and (3) embryo metabolism and cell viability pathways appear to be closely linked, with significant interplay, both between the noted pathways and with the egg microenvironment (Fig. 11). In terms of egg storage protocols, minimal transcriptome differences were found between the C2 and SR groups, indicating that SPIDES treatment recovers most normal early embryonic gene expression despite exposure to prolonged storage. Further studies will be necessary to determine whether gene expression profiles between these two groups remain similar during the remainder of embryonic development. Several studies have documented the impact of prolonged storage carries throughout embryonic development, with metabolic differences and slowed embryonic development following prolonged egg storage [21, 22].

Blastoderm metabolism is minimized through exposure of eggs to cold storage; however, metabolism is not completely halted by cold storage. Additionally, the working hypothesis to explain the molecular mechanisms behind the hatchability benefits of SPIDES treatment is that SPIDES increases embryo metabolism to allow for basic cellular homeostasis mechanisms [23]. Two major metabolic pathways were significantly impacted by experimental treatment: the TCA cycle and oxidative phosphorylation. The two closely linked pathways occur in the mitochondria, with key oxidative phosphorylation genes encoded through the maternally inherited mtDNA, and work together to oxidize acetyl-CoA derived from fat, carbohydrate, and/or protein sources to release stored energy. Both pathways show downregulation in the NSR group, with upregulation in the CR, SR, and C2 groups (Figs. 4 and 9). Further, the NSR group exhibited enrichment for genes involved in the cellular response to glucose starvation, indicating severe impairment in embryonic metabolism (Fig. 2B). Metabolite inputs into the TCA cycle appear to differ between the CR, SR, and C2 treatment groups, potentially due to the advanced developmental age of the SR and C2 groups. The CR group exhibited enrichment of nitrogen compound metabolism specifically related to pyrimidine metabolism (Fig. 2A). Pyrimidines exhibit the highest concentration in the albumen and can be catabolized to intermediates utilized by the TCA cycle, potentially indicating a shift in metabolism [24]. The SR and C2 groups, on the other hand, exhibited upregulation of genes related to beta-oxidation. The SR group displayed upregulation of palmitoyl-coenzyme A binding, which participates in beta-oxidation to yield acetyl-coenzyme A for entry into the TCA cycle (Fig. 8B). Palmitic acid is the major saturated fatty acid in the egg yolk and LDL is the largest lipoprotein component of the egg yolk, which could indicate differential nutrient utilization due to treatment [25].

The mitochondrial genome is essential for oxidative phosphorylation to occur. All 13 of the protein coding genes encoded by mtDNA show significant differential regulation in the four treatment groups, with 11 of the 13 genes exhibiting upregulation in the SR and C2 groups, downregulation in the NSR group, and CR group expression in the middle. Replication of mtDNA to a certain threshold has been shown to be vital during blastoderm development, with unattained thresholds resulting in cell death [26]. *TWNK,* DNA topoisomerase II alpha (***TOP2A***), and DNA polymerase gamma catalytic subunit (***POLG***), which all play key roles in mtDNA replication, were significantly downregulated in the NSR group compared to the SR group, with *TWNK* also identified as the top upstream regulator inhibited in the NSR group in comparisons with both the CR and SR groups (Fig. 7A**,** Table 3, and Table 4). Aside from generating energy for cellular processes and metabolism byproducts that impact egg component composition, TCA cycle metabolites and mtDNA replication have been implicated in the regulation of DNA methylation status in blastocysts from several mammalian species, ultimately regulating downstream gene expression [27, 28].

Byproducts of embryonic metabolism result in increased CO_2_ and ammonia, and ROS production, which can damage the early developing embryo and the surrounding components of the egg. All of the aforementioned metabolic pathways result in CO_2_ and ROS production, with pyrimidine metabolism also producing ammonia. Both the SR and C2 groups exhibited enrichment of lysosome pathways, which aid in recycling cellular waste and the byproducts of energy metabolism (Fig. 4). CO_2_ production within the egg decreases albumen pH (prior to gas exchange through eggshell pores) and ammonia increases albumen pH. Reduced hatchability following prolonged storage is hypothesized to result, in part, due to albumin pH levels too high to successfully support embryonic development [29]. The NSR group exhibited increased expression of solute carrier family 4 member 7 (***SLC4A7***), which is activated in response to increased intracellular pH levels (Fig. 7A) [30]. With prolonged exposure to the increased albumin pH levels, protective barriers for the yolk and blastoderm are broken down, leading to altered yolk lipid concentrations and blastoderm gene expression [23, 31]. In the current study, during blastoderm isolation in the NSR group, it was noted that the perivitelline layer was more fragile than compared to the other experimental groups. In relation to the impact of SPIDES on egg components, it is hypothesized that the intermittent periods of increased cell metabolism produce increased CO_2_ levels which help to buffer the albumen pH increases to a level more ideal for embryonic development, and in turn, protect yolk lipids and the early embryo from the impacts of continued exposure to increased albumen pH levels associated with prolonged storage [32].

Accumulation of ROS causes DNA/RNA damage, which, in turn, impacts DNA-damage-induced cell cycle progression checkpoints and initiates cell apoptosis pathways [33, 34]. NF-E2 related factor 2 (***NFE2I2***), which positively regulates ROS metabolism, was a common predicted upstream regulator in all experimental comparisons, with predicted inhibition in the NSR group (Fig. 9C). Moreover, dihydrofolate reductase (***DHFR***), which was a common DEG amongst all experimental comparisons, plays a role in the removal of superoxide radicals and shows decreased expression in the NSR group in comparison to the other experimental groups (Fig. 1C, D, 10B). Previous studies have also noted differences in oxidative stress genes between blastoderms exposed to prolonged storage and SPIDES treatment [23]. HMBG1 signaling was identified as a common canonical pathway across three experimental comparisons, with HMBG1 signaling activated in the NSR group. High-mobility group box 1 protein (***HMBG1***) is a redox sensitive regulator of apoptosis, further indicating oxidative stress in the NSR group [35]. In this study, the SR group exhibited upregulation of several genes involved in DNA repair mechanisms, such as damage specific DNA binding protein 1 (***DDB1***), replication protein A2 (***RPA2***), replication factor C subunit 5 (***RFC5***), pre-mRNA processing factor 19 (***PRPF19***), and ribosomal L1 domain containing 1 (***RSL1D1***), in the comparison with the NSR group (Fig. 7A). Increased oxidative stress and reduced clearance of ROS in the NSR group may lead to greater DNA/RNA damage, inducing apoptotic pathways, whereas upregulation of DNA repair mechanisms in the SR group may lead to reduced nucleotide damage and reduced downstream apoptotic activation. Aside from DNA damage, increased ROS production can lead to increased lipid peroxidation and is associated with upregulation of TP53-mediated spermidine/spermine N1-acetyltransferase 1 (***SAT1****)* expression, as is seen in the NSR group (Fig. 10A). Yolk lipid peroxidation has been previously associated with prolonged storage and could play a role in the ability of blastodermal cells to resume development [36, 37].

It is well established that prolonged egg storage causes cell cycle arrest and increased apoptosis and necrosis, though the preceding molecular mechanisms are less characterized [38]. Within this dataset, the NSR group exhibits upregulation of genes related to TGF-beta signaling pathway and K48-linked protein ubiquitination pathway, both of which induce apoptosis (Figs. 2B and 4). Within the TGF-beta signaling pathway, the upregulated genes were associated with the cell cycle arrest and apoptosis aspects of the pathway, with increased expression of SMAD specific E3 ubiquitin protein ligase 1 and 2 (***SMURF1*** and ***SMURF2***), E2F transcription factor 4 (***E2F4***), and S-phase kinase associated protein 1 (***SKP1***) in the NSR group compared to the SR group, indicating a potential mechanism for the apoptosis associated with prolonged storage. Similarly, increased TGF beta signaling was also seen in growth arrested cattle blastocysts [39]. K48-linked protein ubiquitination is essential for signaling proteasomal degradation of proteins, with protein ubiquitination triggering apoptosis through TP53 signaling. Moreover, several genes linking protein ubiquitination and apoptosis were predicted, activated upstream regulators of NSR gene expression profiles, such as BARD1 [40].

The NSR group also exhibited predicted inhibition of cell cycle progression and of MYC, an upstream regulator that plays a significant role in the regulation of cell cycle progression, when compared with CR and SR experimental groups (Fig. 6B and 9C). Additionally, MYC is negatively regulated by TP53, which was predicted as an activated upstream regulator in the NSR group and leads to downstream apoptosis (Fig. 9C) [41]. Lastly, the NSR group also showed predicted inhibition of RPL23A, with RPL23A expression required to inhibit TP53 induced apoptosis (Fig. 9B). RPL23A also plays a role in the G_2_/M cell cycle checkpoint, whereas the SR group showed increased expression of S100A6, which is required for progression past the G_2_/M cell cycle checkpoint. (Fig. 10B) [42, 43]. Increased apoptosis mediated through TP53 signaling, due to the combination of reduced cellular metabolism, buildup of metabolism byproducts, DNA damage, cell cycle arrest, and protein ubiquitination, in blastoderms exposed to prolonged storage could ultimately result in blastoderm cell numbers below the required threshold for resumption of development. Gene expression profiles of the SR group were associated with mitigation of several of these features and may allow for blastoderm cell numbers to remain above the required threshold for resumption of development. Interestingly, TP53 signaling was enriched in the SR group, only when compared to the C2 group, indicating that the described rescue mechanisms do not completely mitigate the negative consequences of prolonged storage.

## Conclusions

This study provided a deeper understanding of the molecular pathways altered by egg storage protocols, particularly from a metabolic, cellular homeostasis, and cell viability perspective. Additional research is needed to experimentally validate the conclusions made from analysis of transcriptome data, especially in relation to potential differences in TCA cycle inputs and metabolites, mtDNA replication levels, and cell cycle arrest between blastoderms exposed to prolonged storage and SPIDES. In this study, prolonged storage was associated with insufficient cellular metabolism and mtDNA replication, cell cycle arrest, and increased protein ubiquitination and apoptotic signaling. SPIDES treatment was associated with increased lipid beta-oxidation and mtDNA replication, cell cycle progression, and increased DNA repair mechanisms. The interplay between these factors and between the developing blastoderm and the microenvironment of the egg is complex, requiring future studies to determine potential interactions and gauge the significance of these interactions for the purpose of egg storage protocol optimization during periods of prolonged storage.

## Methods

### Incubation and Blastoderm isolation

This research was approved by the USDA Beltsville Agricultural Research Center (**BARC**) Animal Care and Use Committee, with all methods carried out in accordance with relevant guidelines and regulations. The study was carried out in compliance with the ARRIVE guidelines. Ross broiler eggs (hen age was 40 weeks) were used for this study. Eggs were obtained from a commercial egg depot and shipped overnight in a refrigerated truck (16 °C) to the BARC hatchery. Eggs were randomly assigned to four treatment groups: CR, C2, NSR, or SR. In the CR group, eggs were stored at 17 °C, undisturbed for 4 days post-oviposition. The CR group served as the industry standard storage method. In the C2 group, eggs were stored at 17 °C, undisturbed for 4 days post-oviposition followed by 10 h of incubation to advance embryonic development to the stage (HH: stage 2) seen previously in embryos following SPIDES treatment [44]. The C2 group served as normal embryonic development following standard industry egg storage. In the NSR group, eggs were stored at 17 °C, undisturbed for 21 days post-oviposition. The NSR group served as storage duration previously associated with the negative consequences of prolonged storage. Lastly, in the SR group, eggs were stored at 17 °C for 21 days post-oviposition and moved to the incubator on days 6, 12, and 18 for a 4-h incubation period each time. The SR group served as the proposed treatment for mitigating the negative effects of prolonged egg storage. Egg storage and incubation were performed as previously described [19]. Briefly, egg storage occurred in a walk-in cold room maintained at 16 to 17 °C with 75% relative humidity (**RH**). Egg incubation occurred at 37.5 °C with 60% RH. Blastoderms were isolated as previously described [45]. Briefly, sterile filter paper rings and utensils were used to transfer the blastoderm from the ovum surface to a sterile PBS constraining petri dish, where white and yellow yolk was carefully removed from the surface of the embryo. Embryos were staged visually using the tables described by Eyal-Giladi and Kochav (**EG&K**) [46] and Hamburger and Hamilton [44] to ensure embryonic development was consistent within an experimental group. Embryonic development stage at isolation was the same for the CR and NSR groups (both groups not exposed to incubation conditions – EG&K stage X). Similarly, embryonic development stage at isolation was the same for C2 and SR groups (both groups exposed to incubation conditions – HH stage 2). A total of 7 isolated blastoderms were pooled per experimental replicate, with three replicates per experimental group (*n* = 3).

#### RNA isolation, cDNA library construction, and sequencing

A RNeasy Mini kit (Qiagen, Valencia, CA) was used to extract total RNA from pooled blastoderm samples. RNA extraction included an on-column deoxyribonuclease digestion. Total concentration of RNA was determined via NanoDrop 2000 spectrophotometer (Thermo Fisher Scientific, USA). RNA quality was determined using an Agilent 2100 Bioanalyzer (Agilent Technology, Santa Clara, CA). RNA integrity number (RIN) values for all samples were above 8.5, with an average RIN value of 9.43. A TruSeq stranded mRNA library prep kit (Illumina, San Diego, CA) was used to generate sequencing libraries from 1 μg total RNA, following the manufacturer’s procedure. Libraries were quantified using an Agilent 2100 Bioanalyzer and High Sensitivity DNA Kit. For sequencing, 3 libraries were pooled per lane (4 lanes total). Pools were submitted to Johns Hopkins Single Cell and Transcriptomics Core facility for paired-end sequencing (100 bp reads) on an Illumina HiSeq 2500.

#### Bioinformatic analysis of sequencing data

All sequencing files were submitted to the NIH Short Read Archive (accession numbers SAMN21906327- SAMN21906338) (https://www.ncbi.nlm.nih.gov/sra). Bioinformatic analysis of sequencing data was performed using CLC Genomics Workbench 20.0 (Qiagen, Valencia, CA; https://digitalinsights.qiagen.com). Quality of raw and trimmed sequencing reads was assessed through FastQC (http://www.bioinformatics.babraham.ac.uk/projects/fastqc/) [47]. Sequences with a Phred quality score less than 20 and adapter sequences were trimmed from raw reads. Reads were mapped to the *Gallus gallus* reference genome (GRCg6a; ENSEMBL annotation release 101; http://uswest.ensembl.org/Gallus_gallus/Info/Index) [48]. DEGs were identified through pairwise comparisons between C2 and CR, NSR and CR, NSR and SR, and C2 and SR groups.

Functional analysis of DEGs was performed using the functional annotation tool of Database for Annotation, Visualization and Integrated Discovery (**DAVID**) v6.8 [49, 50] (including KEGG pathway analysis) [51–53] and QIAGEN Ingenuity Pathway Analysis (**IPA**) (Qiagen, Valencia, CA; https://www.qiagenbioinformatics.com/products/ingenuity- pathway-analysis) [54]. Functional analysis was only performed for DEGs with a *q-*value less than 0.05, an absolute fold change greater than 1.5, and a FPKM value greater than 20. The distribution of log_2_ transformed FPKM values was examined across all comparisons to determine the appropriate FPKM cutoff to work within the recommended number of DEGs inputted for analysis with DAVID and IPA. For analysis of gene ontology terms through DAVID, only terms with a gene count greater than 5 and a *P-*value less than 0.05 were considered. For analysis of canonical pathways, upstream regulators, and causal networks through IPA, an absolute z-score greater than 2 and *P-*value less than 0.05 were considered to be significantly activated or inhibited. For regulator effects in IPA, only effects with a positive consistency score, an absolute z-score greater than 2, and a *P-*value less than 0.05 were analyzed. For networks in IPA, only networks with scores greater than 40 were considered.

#### Confirmation RNA sequencing results by RT-qPCR

DEGs selected for RNAseq confirmation fit the following parameters: *q* < 0.05, absolute fold change greater than 1.5, annotated in the chicken genome, encoded by a single transcript, and a FPKM value greater than 20. Gene expression results as determined by RNA sequencing were confirmed through custom profiler quantitative PCR arrays, as previously described [23]. Briefly, one microgram of total RNA from each pooled blastoderm sample was converted to cDNA using RT^2^ First Strand Kit (Qiagen, Valencia, CA). A Custom RT^2^ Profiler PCR Array (Qiagen, Valencia, CA) using chicken specific primers was used to confirm RNA sequencing gene expression results. All reactions were run in duplicate and were carried out using CFX96 Real-Time PCR system (Bio-Rad, Hercules, CA), with the following cycling conditions: 95 °C for 10 min, followed by 40 cycles at 95 °C for 15 s, 60 °C 1 min. Levels of mRNA were assessed for ABL proto-oncogene 1, non-receptor tyrosine kinase (***ABL1***), hypoxanthine phosphoribosyltransferase 1 (***HPRT1***), TATA-box binding protein (***TBP***), cytoglobin (***CYGB***), albumin (***ALB***), non-POU domain containing, octamer-binding (***NONO***), glucuronidase beta (***GUSB***), *TP53*, phosphoglycerate kinase 2 (***PGK2***), and myoglobin (***MB***). Gene expression data were normalized to the averaged expression of beta-actin (***ACTB***), beta-2-microglobulin (***B2M***), and glyceraldehyde 3-phosphate dehydrogenase (***GAPDH***) and analyzed via the delta-delta Ct method. Data are presented relative to average CR mRNA levels for each gene examined. Data were normalized, log_2_ transformed, and analyzed by a one-way ANOVA using SAS software (SAS Institute, Cary, NC). The PDIFF statement was used to compare the least squares means.

## Supplementary Information

**Additional file 1.** Mapping and fragment statistics. (A) The number of reads obtained for each sample, with the proportions of reads mapped in pairs, reads mapped in broken pairs, and reads not mapped illustrated. (B) The number of mapped fragments obtained for each sample, with the proportions of fragments mapping to exonic, intronic, and intergenic regions illustrated.

**Additional file 2.** Principle component and heat map analysis. (A) Principle component analysis plot illustrating grouping of individual samples for each experimental group. (B) Heat map analysis illustrating grouping of individual samples for each experimental group.

**Additional file 3 **Confirmation of RNA sequencing results. Confirmation by quantitative PCR of gene expression as determined by RNA sequencing. Data are presented relative to control (CR) for each gene. (A) Levels of mRNA obtained through quantitative PCR (circle) for ABL proto-oncogene 1, non-receptor tyrosine kinase (***ABL1***), hypoxanthine phosphoribosyltransferase 1 (***HPRT1***), TATA-box binding protein (***TBP***), cytoglobin (***CYGB***), albumin (***ALB***), non-POU domain containing, octamer-binding (***NONO***), glucuronidase beta (***GUSB***), tumor protein p53 (***TP53***), phosphoglycerate kinase 2 (***PGK2***), and myoglobin (***MB***) are shown overlaid on FPKM values obtained through RNA sequencing. (B) Group averages, standard deviation, and *p*-values for each gene for both quantitative PCR and RNA sequencing approaches, with letters denoting significant expression differences between experimental groups. Significant *p*-values are highlighted in gray for comparison of approaches.

**Additional file 4.** Differential expression data for experimental comparisons. For each comparison, differential expression output from CLC Genomics Workbench is presented.

**Additional file 5.** Top expressed transcripts. FPKM values for the top 20 transcripts exhibiting the highest expression in each experimental group. Gene symbols are color coded to show the degree of overlap among the experimental groups. The total number of transcripts exhibiting a FPKM value over 20 for each experimental group is also shown.

## Data Availability

The datasets supporting the conclusions of this article are available in the NCBI Short Read Archive (SRA; https://www.ncbi.nlm.nih.gov/sra) repository, accession numbers, SAMN21906327- SAMN21906338. Additionally, datasets supporting the conclusions of this article are included within the article (Additional file 4).

## Abbreviations

*ABL1*: ABL proto-oncogene 1, non-receptor tyrosine kinase
*ACTB*: Beta-actin
*ALB*: Albumin
AREG: Amphiregulin
*B2M*: beta-2-microglobulin
BAG1: BAG cochaperone 1
BARC: Beltsville Agricultural Research Center
BARD1: BRCA1 associated RING domain 1
BIRC5: Baculoviral IAP repeat containing 5
BP: Biological process
C2: Incubated control group
CC: Cellular component
CCNF: Cyclin F
CO_2_: Carbon dioxide
CR: Control group
*CYGB*: Cytoglobin
DAVID: Database for Annotation, Visualization and Integrated Discovery
*DDB1*: Damage specific DNA binding protein 1
DEG: Differentially expressed gene
*DHFR*: Dihydrofolate reductase
*E2F4*: E2F transcription factor 4
EG&K: Eyal-Giladi and Kochav
FPKM: Fragments per kilobase of exon per million reads mapped
*GAPDH*: Glyceraldehyde 3-phosphate dehydrogenase
GO: Gene ontology
*GUSB*: Glucuronidase beta
HH: Hamburger–Hamilton
*HMBG1*: High-mobility group box 1 protein
*HPRT1*: Hypoxanthine phosphoribosyltransferase 1
IPA: Ingenuity Pathway Analysis
MACROH2A1: macroH2A.1 histone
MAP2K4: mitogen-activated protein kinase kinase 4
*MB*: Myoglobin
MF: Molecular function
MXD1: MAX dimerization protein 1
MYC: MYC proto-oncogene, BHLH transcription factor
*NFE2I2*: NF-E2 Related Factor 2
NFKBIA: NFKB inhibitor alpha
*NONO*: non-POU domain containing, octamer-binding
NSR: Prolonged storage group
PA2G4: Proliferation-associated 2G4
PDGF BB: Platelet derived growth factor subunit B
*PGK2*: Phosphoglycerate kinase 2
*POLG*: DNA polymerase gamma catalytic subunit
*PRPF19*: Pre-MRNA Processing Factor 19
RABL6: RAB, member RAS oncogene family like
*RFC5*: Replication factor C subunit 5
RH: Relative humidity
ROS: Reactive oxidative species
*RPA2*: Eplication protein A2
RPL23A: Ribosomal protein L23a
RSL1D1: Ribosomal L1 domain containing 1
*SAT1*: Spermidine/spermine N1-acetyltransferase 1
*SKP1*: S-phase kinase associated protein 1
*SLC4A7*: Solute carrier family 4 member 7
*SMURF1*: SMAD specific E3 ubiquitin protein ligase 1
*SMURF2*: SMAD specific E3 ubiquitin protein ligase 2
SOX17: SRY (Sex Determining Region Y)-Box 17
SPIDES: Short periods of incubation during egg storage
SR: SPIDES group
*TBP*: TATA-box binding protein
TCF3: Transcription factor 3
*TOP2A*: DNA Topoisomerase II Alpha
*TP53*: Tumor protein p53
TSC22D1: TSC22 domain family member 1
TWNK: Twinkle mtDNA helicase
XBP1: x-box binding protein 1
