# Supplementary figures and images for: Transcriptome analysis of blastoderms exposed to prolonged egg storage and short periods of incubation during egg storage
Source: BMC Genomics. 2022 Apr 4;23:262. doi: 10.1186/s12864-022-08463-2 (PMC8981843; doi:10.1186/s12864-022-08463-2)

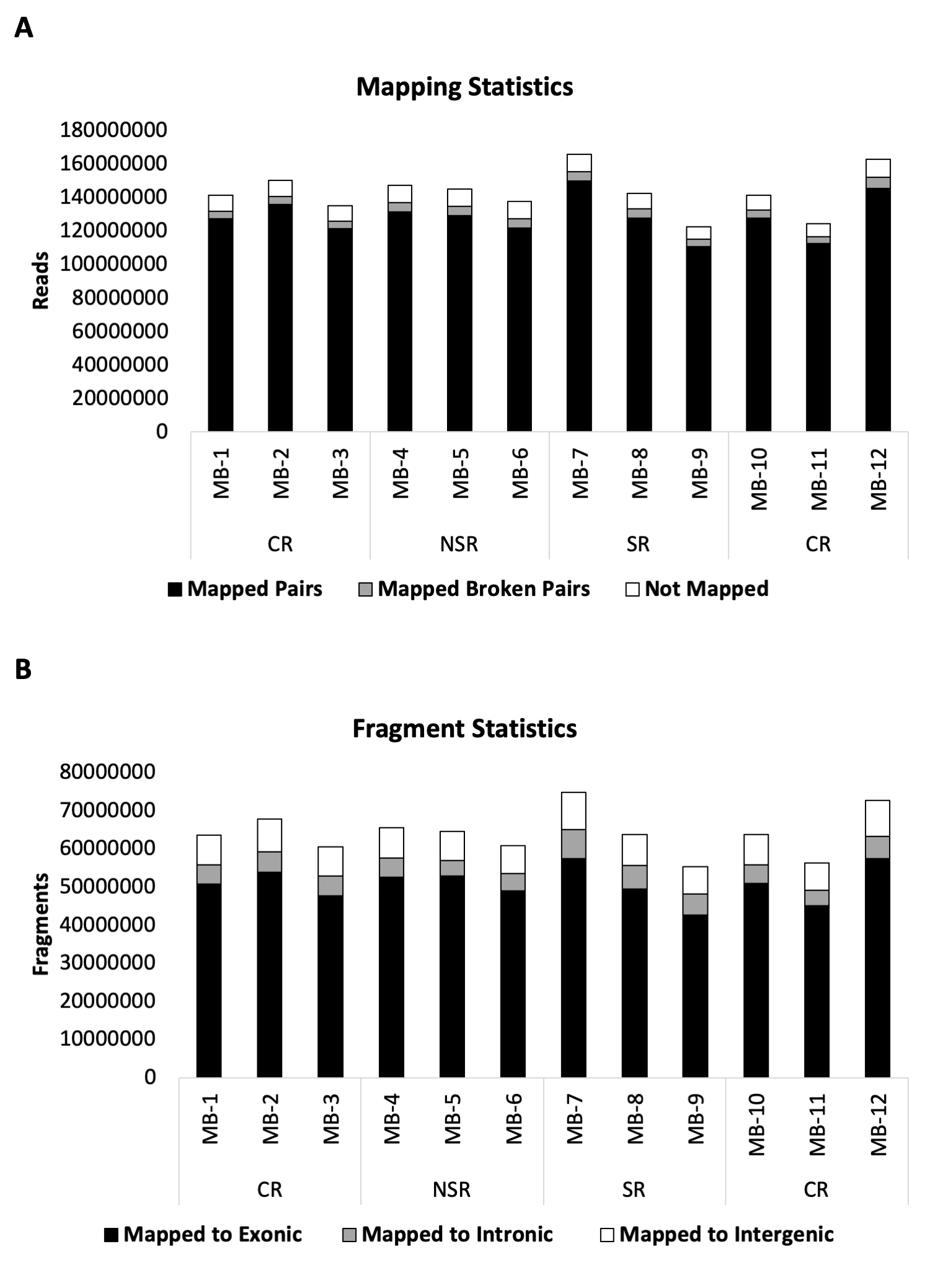

Supplement: Supplementary file 1 — Additional file 1. Mapping and fragment statistics. (A) The number of reads obtained for each sample, with the proportions of reads mapped in pairs, reads mapped in broken pairs, and reads not mapped illustrated. (B) The number of mapped fragments obtained for each sample, with the proportions of fragments mapping to exonic, intronic, and intergenic regions illustrated. [file 12864_2022_8463_MOESM1_ESM.tiff]

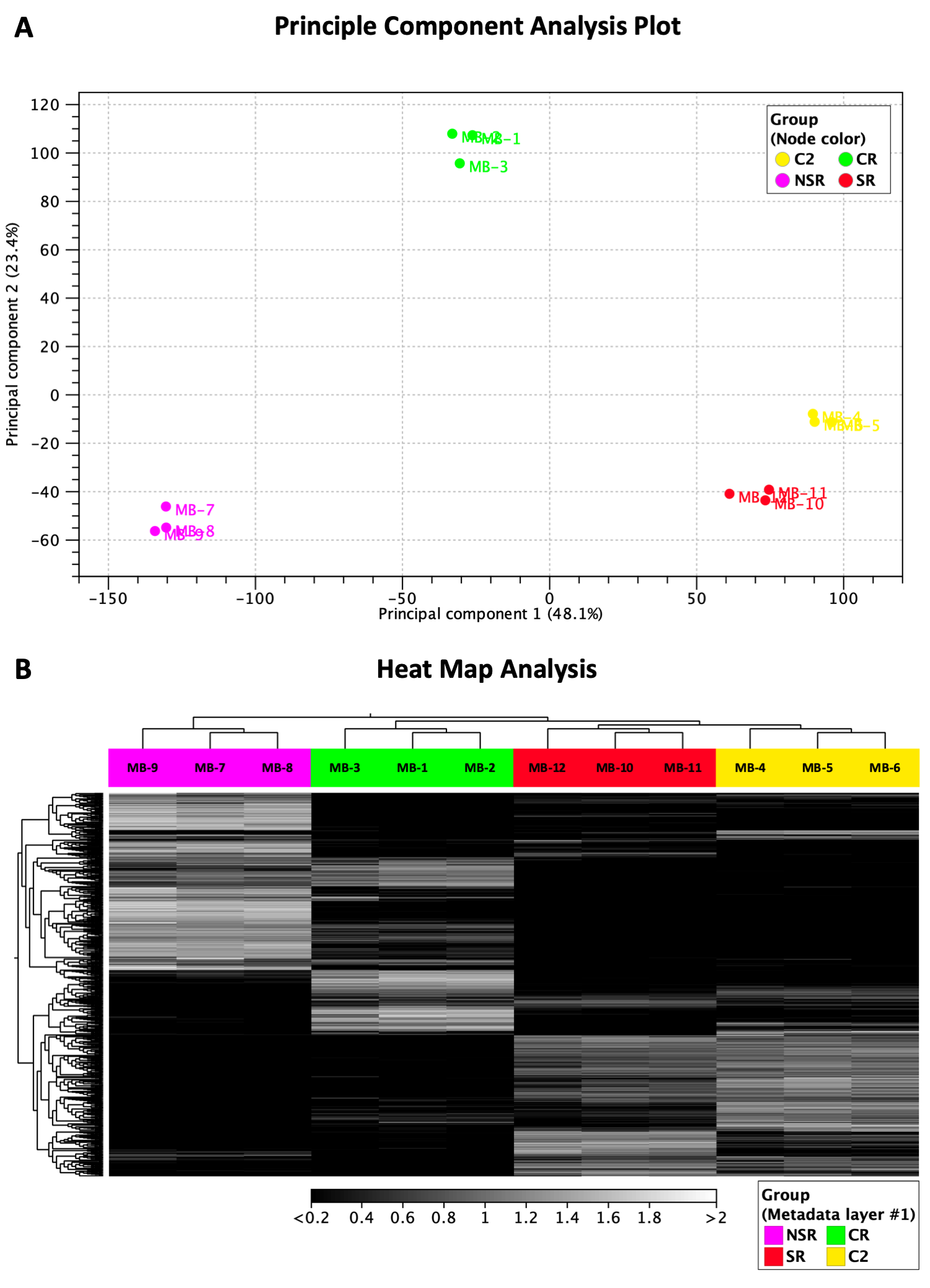

Supplement: Supplementary file 2 — Additional file 2. Principle component and heat map analysis. (A) Principle component analysis plot illustrating grouping of individual samples for each experimental group. (B) Heat map analysis illustrating grouping of individual samples for each experimental group. [file 12864_2022_8463_MOESM2_ESM.tiff]

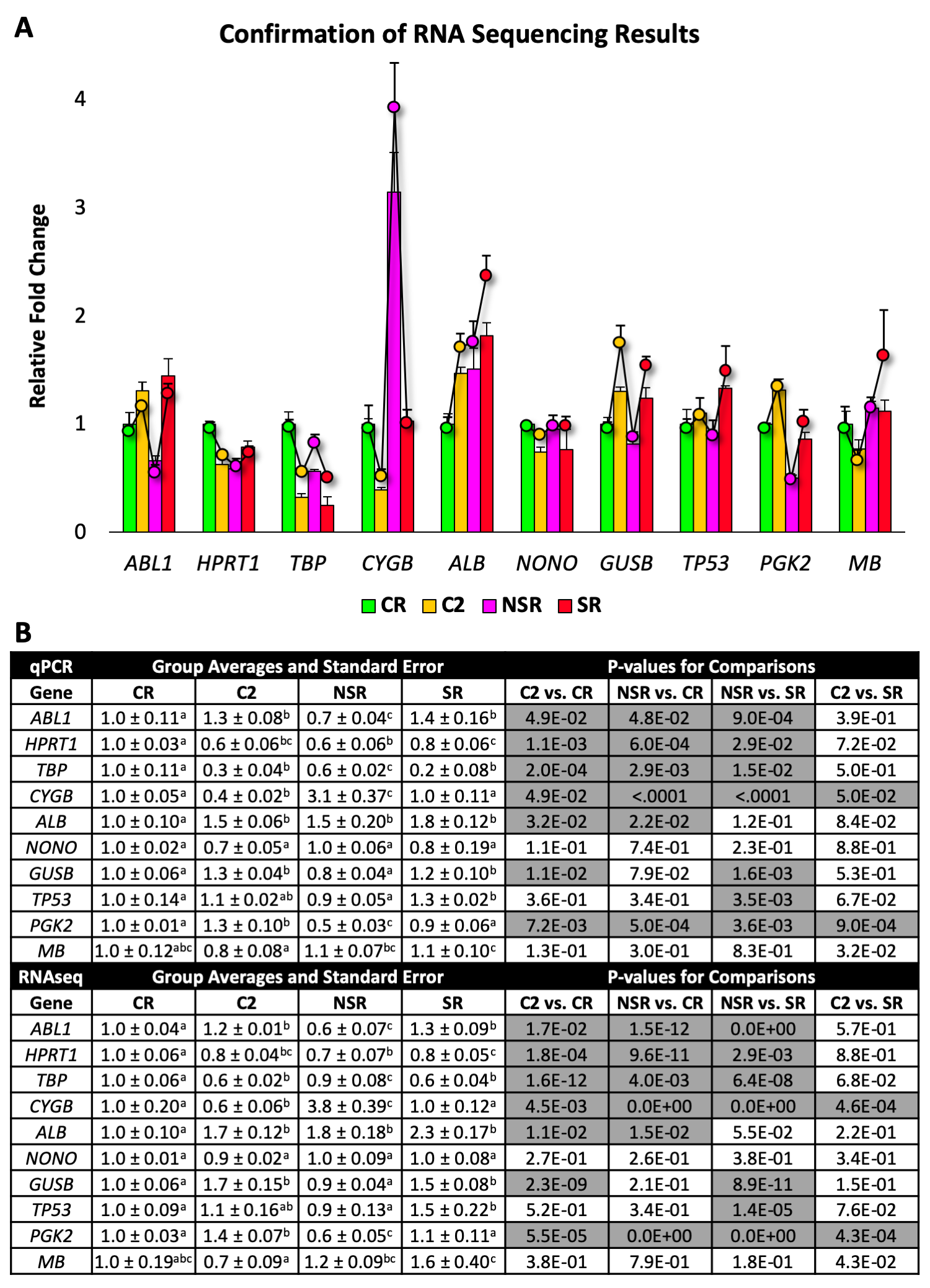

Supplement: Supplementary file 3 — Additional file 3 Confirmation of RNA sequencing results. Confirmation by quantitative PCR of gene expression as determined by RNA sequencing. Data are presented relative to control (CR) for each gene. (A) Levels of mRNA obtained through quantitative PCR (circle) for ABL proto-oncogene 1, non-receptor tyrosine kinase (ABL1), hypoxanthine phosphoribosyltransferase 1 (HPRT1), TATA-box binding protein (TBP), cytoglobin (CYGB), albumin (ALB), non-POU domain containing, octamer-binding (NONO), glucuronidase beta (GUSB), tumor protein p53 (TP53), phosphoglycerate kinase 2 (PGK2), and myoglobin (MB) are shown overlaid on FPKM values obtained through RNA sequencing. (B) Group averages, standard deviation, and p-values for each gene for both quantitative PCR and RNA sequencing approaches, with letters denoting significant expression differences between experimental groups. Significant p-values are highlighted in gray for comparison of approaches. [file 12864_2022_8463_MOESM3_ESM.tiff]
